# Investigation of Studies on ChatGPT's Ability to Answer Anatomy Questions: A Self-Evaluation by ChatGPT and Comparison with an Evaluation by Gemini

**DOI:** 10.7759/cureus.90572

**Published:** 2025-08-20

**Authors:** Dimitrios Chytas, George Noussios, Marios Salmas, Angelo V Vasiliadis, Theodore Troupis

**Affiliations:** 1 Physiotherapy, University of Peloponnese, Sparta, GRC; 2 Physical Education and Sports Sciences (Serres), Aristotle University of Thessaloniki, Thessaloniki, GRC; 3 Anatomy, National and Kapodistrian University of Athens School of Medicine, Athens, GRC; 4 Sports Trauma and Orthopaedics, St. Luke’s Hospital, Thessaloniki, GRC; 5 Surgery, University of Athens Medical School, Athens, GRC

**Keywords:** anatomy, anatomy education, artificial intelligence, chatgpt, gemini, large language models

## Abstract

Introduction: There is controversy about ChatGPT’s (OpenAI, San Francisco, CA) potential to answer anatomy questions and play a significant role in anatomy education. We aimed to assess ChatGPT’s ability to locate and summarize literature about its performance in answering anatomy questions. We also aimed to explore Gemini’s (Google, Mountain View, CA) ability to perform this task.

Methods: We asked ChatGPT and Gemini to list and summarize five studies: 1) about ChatGPT’s ability to answer anatomy questions, 2) comparing ChatGPT with another artificial intelligence (AI) tool in terms of answering anatomy questions, and 3) showing that ChatGPT answered anatomy questions with success lower than 70%. For each query, we measured how many studies were 1) correctly identified and 2) accurately summarized, and we assessed the presence of any bias.

Results: ChatGPT performed excellently in the first query (100%/100%), poorly in the second (20%/20%), and moderately in the third query (60%/40%). It conducted a strict self-evaluation and did not hallucinate. Gemini’s performance was 100%/40%, 60%/40%, and 80%/60% in the three queries, respectively. It was inflexible in finding a variety of publications, while it often hallucinated and exhibited bias against ChatGPT.

Conclusions: ChatGPT and Gemini generated reliable responses only when they were simply asked to detect studies about ChatGPT’s ability to answer anatomy questions. They particularly struggled to locate and outline comparative studies. While Gemini performed generally better, it hallucinated and exhibited bias against ChatGPT. Ongoing evolution may enhance ChatGPT and Gemini’s potential to contribute to anatomical research.

## Introduction

Artificial intelligence (AI) has recently gained enhanced research interest in several scientific fields, with anatomy education being among them [1]. Much of this interest has focused on ChatGPT (OpenAI, San Francisco, CA), which currently lacks evidence to support a significant role in anatomy teaching [1]. Nevertheless, ChatGPT’s rapidly evolving technology may soon position it as a valuable educational tool [1].

Especially concerning ChatGPT’s answers to anatomical questions, there are conflicting outcomes in the literature. Saluja and Tigga [2] concluded that ChatGPT can contribute to students’ understanding of the features and clinical significance of anatomical structures, generate sufficient outlines of lectures, create questions, and assess students’ answers, providing adequate reasoning. Of note, Talan and Kalinkara [3] compared ChatGPT’s performance in anatomical questions with that of health sciences students, who had received a four-week course. ChatGPT outperformed students, answering correctly 67.5% of questions, versus 52.8% that were correctly answered by the students. Interestingly, in the study by Collins et al. [4], ChatGPT’s responses to multiple-choice anatomy questions were 100% accurate, while in the study by Ilgaz and Çelik [5], its performance did not surpass 50%.

On the other hand, Gemini (Google, Mountain View, CA) is another AI platform that has not received as much scientific interest as ChatGPT in the domain of anatomy education. Similar to ChatGPT, conflicting outcomes have been reported regarding its anatomy teaching potential. In the paper by Ganapathy and Kaushal [6], ChatGPT-4o mini achieved a significantly better score (76.15%) than Gemini (72.84%). Although in application-level questions ChatGPT-4o mini was superior to Gemini, the latter performed better in comprehension-level questions (76.88% vs. 73.66%). Despite the substantial body of research regarding the potential use of ChatGPT in anatomy education [1], there is a lack of studies to evaluate ChatGPT’s ability to locate and outline literature about its performance in answering anatomy questions. Thus, we aimed to investigate this ability and compare it with that of Gemini. We also aimed to detect any bias in the answers generated.

## Materials and methods

Study design

Five anatomy teachers collaborated to design three queries that asked ChatGPT to find literature about its ability to answer anatomy questions. Our purpose was 1) to explore ChatGPT’s ability to evaluate its performance via finding relevant literature, 2) to compare this ability with that of Gemini, and 3) to detect any bias in the two platforms’ generated text. Our exploration involved three levels: the two AI platforms’ general ability to find literature about ChatGPT’s performance in anatomy questions; 2) their ability to find studies including comparisons of ChatGPT with other AI platforms in terms of their performance; and 3) their ability to quantitatively assess ChatGPT’s performance in anatomy questions.

Queries

The three queries were as follows: "Please list five peer-reviewed studies about the ability of ChatGPT to answer anatomy questions. Please summarize their results."2) "Please list five peer-reviewed studies that compared ChatGPT with another artificial intelligence tool in terms of answering anatomy questions. Please summarize their outcomes, and do not repeat previously mentioned studies."3) "Please list five peer-reviewed studies that showed that ChatGPT answered anatomy questions with a success lower than 70%. Please summarize their outcomes and do not repeat previously mentioned studies." The two accessed platforms were ChatGPT-4.0 Turbo (https://chatgpt.com) and Gemini 2.5 Flash (https://gemini.google.com). These queries were submitted to both platforms on July 10, 2025. 

Evaluation of responses

Each author independently assessed how many of the five required studies were successfully retrieved and accurately summarized by ChatGPT and Gemini at each of the three queries. We calculated the percentage of successful detection and accurate summarization in each case. We defined “accurate summary” as the summary that contained true information about all the relevant outcomes of the article’s abstract. When the published abstract included numerical data, its mention was considered necessary. The simple description of outcomes without the accompanying numerical data was considered inadequate to make the generated summary “accurate.” The answers given by each platform were qualitatively assessed. The five anatomy teachers discussed performing their final evaluation. In case of disagreement, the decision was made by the senior author.

## Results

In all three queries, each platform provided the titles and summaries of five studies. ChatGPT additionally provided links to the corresponding published titles and abstracts. The responses are outlined in Table 1.

**Table 1:** Summary of answers given by ChatGPT and Gemini

Query	ChatGPT	Gemini
Please list five peer-reviewed studies about the ability of ChatGPT to answer anatomy questions. Please summarize their results.	ChatGPT successfully identified all five required studies (100%) and accurately summarized them (100%).	Gemini successfully identified all five required studies (100%) but accurately summarized only two of them (40%). Two cases of inaccuracy against ChatGPT.
Please list five peer-reviewed studies that compared ChatGPT with another artificial intelligence tool in terms of answering anatomy questions. Please summarize their outcomes and do not repeat previously mentioned studies.	ChatGPT correctly identified only one of the five required studies (20%), which was accurately summarized (20%).	Gemini successfully identified three of the five required studies (60%). From the three correctly identified studies, two were accurately summarized (40%). One case of inaccuracy against ChatGPT (and simultaneously in favor of Gemini).
Please list five peer-reviewed studies that showed that ChatGPT answered anatomy questions with a success lower than 70%. Please summarize their outcomes and do not repeat previously mentioned studies.	ChatGPT correctly detected three of the five required studies (60%) and accurately summarized two of the five required studies (40%).	Although Gemini correctly detected four of the five required studies (80%), all these studies were previously mentioned, despite the instruction. The platform accurately summarized three of the five required studies (60%). One (repeated) case of inaccuracy against ChatGPT.

ChatGPT’s response to the first query

The review by Chytas et al. [1] showed that ChatGPT’s answers to anatomy questions were unsatisfactory. Despite this, ChatGPT helped students grasp anatomical concepts and clinical relevance. When compared, it performed worse or similarly to other platforms. In the study by Bolgova et al. [7], ChatGPT 3.5 accurately answered 58.4% of questions regarding the back and 48.8% of head and neck questions, but its performance concerning vessels and nerves was poor. Mavrych and Bolgova [8] found that the success of ChatGPT‑3.5 and ChatGPT‑4 in answering thoracic anatomy questions was 45.6% and 75.6%, respectively. Mantzou et al. [9] showed that 50% of the answers of ChatGPT-3.5 to musculoskeletal anatomy questions were of "good quality" (based on a five-point Likert scale), and the consistency across time points was 66.6%. Some responses had significant errors or conflicting information. In the paper by Sarangi et al. [10], ChatGPT‑4’s success in answering radiologic anatomy questions, with and without exam‑style context, was 7.5% and 4%, respectively (Table 2).

**Table 2:** Summary of ChatGPT’s responses to the first query

Study	Key points
Chytas et al. [1]	ChatGPT helped students grasp anatomical concepts and clinical relevance. However, answers lacked detail, image generation was poor, and texts were not scientifically robust.
Bolgova et al. [7]	ChatGPT answered correctly about 58% of questions about the back, and about 49% of head and neck questions. The performance was poor in vascular/nerve questions.
Mavrych and Bolgova [8]	ChatGPT‑3.5 provided about 45.6% accurate answers to thoracic anatomy questions, while ChatGPT‑4’s accuracy was about 75.6%.
Mantzou et al. [9]	ChatGPT‑3.5 answered musculoskeletal anatomy questions at four different times; three out of six answers were rated “good” or better. Consistency across time was 67%.
Sarangi et al. [10]	ChatGPT‑4 answered radiologic anatomy questions. The correct identification was 4% (without context) versus 7.5% (with context).

Gemini’s response to the first query

Mavrych et al. [11] did not include numerical results but showed strengths and weaknesses of ChatGPT compared to Copilot, PaLM, Bard, and Gemini, and that no single model is definitively superior to others. In the paper by Bolgova et al. [7], ChatGPT-3.5 accurately answered 44.1% of multiple-choice anatomy questions. The percentages concerning back, head/neck, pelvis, thorax, and upper limb were 58.4%, 48.8%, 45.6%, 37.6%, and 36.4%, respectively. The platform struggled with questions about blood supply and innervation. Shamith et al. [12] (incorrectly cited as “Adams et al.”) found that ChatGPT had limitations in generating anatomical images and identifying structures from foot images. Arun et al. [13] showed that Anatbuddy, a customized AI model, could outperform ChatGPT regarding factual accuracy and relevance for specific anatomical queries. Mantzou et al. [9] (misattributed to “Totlis et al.”) found that about 50% of ChatGPT-3.5’s answers to musculoskeletal anatomy questions were rated as "good quality" (scoring ≥3 on a five-point Likert scale), and the consistency across time points was around 66.6%. There were significant mistakes, conflicting data, or a lack of information in low-quality responses (Table 3).

**Table 3:** Summary of Gemini’s responses to the first query

Study	Key points
Mavrych et al. [11]	There are strengths and weaknesses of ChatGPT in relation to other platforms.
Bolgova et al. [7]	ChatGPT is a promising tool but has limitations, particularly with more complex and detailed anatomical information.
Shamith et al. [12]	There are limitations in ChatGPT's visual anatomical knowledge and its ability to accurately generate or interpret anatomical images.
Arun et al. [13]	Anatbuddy could potentially outperform ChatGPT in terms of factual accuracy and relevance for specific anatomical queries.
Mantzou et al. [9]	ChatGPT made significant mistakes. It is not yet a consistently reliable, independent resource for learning musculoskeletal anatomy.

ChatGPT’s response to the second query

Mavrych et al. [11] found that ChatGPT‑4 had better performance in answering anatomy questions than ChatGPT‑3.5, Bard, Copilot, PaLM, and Gemini. In the paper by Al-Sharif et al. [14], ChatGPT and Bard answered patients' oculoplastics-related questions. ChatGPT excelled in answering lacrimal questions (83.8%), while Bard performed best in the questions about eyelids (60.4%). Anvari et al. [15] showed that ChatGPT‑4 had the highest accuracy in answering hepatology questions, followed by Bing (based on ChatGPT‑4) and then Bard. Bolgova et al. [16] found that the performance of ChatGPT‑4, Gemini, and Copilot in biochemistry questions was 85%, 78.5%, and 64%, respectively. ChatGPT‑4 was superior to other platforms for preclinical science, including anatomical content. In the study by Mavrych et al. [17], Claude achieved the best score in neuroscience questions, followed by ChatGPT‑4, Copilot, ChatGPT‑3.5, and Gemini (Table 4).

**Table 4:** Summary of ChatGPT’s responses to the second query

Study	Key points
Mavrych et al. [11]	ChatGPT‑4 provided more accurate answers than ChatGPT‑3.5, Bard, Copilot, PaLM, and Gemini.
Al-Sharif et al. [14]	ChatGPT outperformed Bard for lacrimal‑system anatomy. Bard did better for eyelid anatomy but was still less accurate overall.
Anvari et al. [15]	ChatGPT‑4 answered hepatology questions with the highest accuracy, followed by Bing, then Bard.
Bolgova et al. [16]	GPT‑4 provided about 85% correct answers to biochemistry questions (consistent across attempts). It performed better than Gemini and Copilot.
Mavrych et al. [17]	Regarding neuroanatomy and neuroscience questions, Claude and GPT‑4 outperformed students, followed by Copilot, GPT‑3.5, and Gemini.

Gemini’s response to the second query

In the study by Al-Khater [18] (incorrectly cited as “Perumal et al.”), ChatGPT demonstrated higher accuracy than Gemini and Claude when answering anatomy questions. However, Claude provided more organized and comprehensive responses. Gemini performed less effectively regarding accuracy and scientific explanation. Wickramarathna et al. [19] (misattributed to “Somasundaram et al.”) found that ChatGPT-3.5 was superior to Bing GPT-4 in providing anatomical information. Both AI tools need improvement, while traditional methods remain superior in accuracy and relevance. Ilgaz and Çelik [5] (incorrectly cited as “Totlis et al.”) found that ChatGPT and Google Bard answered anatomy questions with scores below 50%, without a statistically significant difference. In the paper by Sallam et al. [20] (misattributed to “Alimova and Barch”), ChatGPT-4 answered significantly better than ChatGPT-3.5, Bing, Bard, and postgraduate students to clinical chemistry questions, a performance highly relevant to these platforms’ potential regarding anatomy. The review by Wang et al. indicates that while ChatGPT shows considerable promise and often outperforms some AI models in answering medical questions (including those on anatomy), its performance can be inconsistent (Table 5).

**Table 5:** Summary of Gemini’s responses to the second query

Study	Key points
Al-Khater [18]	ChatGPT demonstrated higher accuracy than Gemini and Claude. However, Claude provided more organized and comprehensive anatomical responses.
Wickramarathna et al. [19]	ChatGPT-3.5 and Bing GPT-4 could provide detailed descriptions of gross anatomy, human embryology, and histology. However, ChatGPT-3.5 was generally superior to Bing GPT-4 in providing anatomical information.
Ilgaz and Çelik [5]	There was no statistically significant difference between ChatGPT and Google Bard in their ability to answer anatomy questions.
Sallam et al. [20]	ChatGPT-4 significantly outperformed ChatGPT-3.5, Bing, Bard, and even human postgraduate students.
Wang et al.	While ChatGPT shows considerable promise and often outperforms some older or general AI models in answering medical questions (including those on anatomy), its performance can be inconsistent.

ChatGPT’s response to the third query

Shah et al. [21] found that ChatGPT-3.5 scored less than 40% in anatomical questions that were asked in the context of hand surgery examinations. Leitão et al. [22] showed that ChatGPT answered correctly at only 20% of anatomy-focused radiologic questions. In the paper by Suárez et al. [23], ChatGPT’s score in clinical endodontics questions was 57.3%. Pavone et al. [24] noted that ChatGPT’s performance was 59% concerning gynecological endoscopic surgical knowledge questions and that it scored well under anatomical knowledge expectations. Siebielec et al. [25] found that ChatGPT‑3.5’s grade in Polish medical exams was approximately 60%, slightly below the typical passing thresholds, and that the performance was under 70% across major medical licensing questions involving anatomy, physiology, and clinical reasoning (Table 6).

**Table 6:** Summary of ChatGPT’s responses to the third query

Study	Key points
Shah et al. [21]	ChatGPT‑3.5 answered hand surgery questions. Concerning anatomy/basic science/imaging, the performance was lower than 40%.
Leitão et al. [22]	ChatGPT answered correctly 20% of anatomy questions.
Suárez et al. [23]	ChatGPT provided 57.3% correct answers to dichotomous clinical questions.
Pavone et al. [24]	ChatGPT provided 59% correct answers to gynecological endoscopic surgical knowledge questions.
Siebielec et al. [25]	ChatGPT‑3.5 answered questions of Polish medical exams with an accuracy of 55%–60%, slightly below typical passing thresholds.

Gemini’s response to the third query

In the studies by Bolgova et al. [7] and Mantzou et al. [9] (misattributed to “Totlis et al.”), the aforementioned data were entirely repeated. In the paper by Shamith et al. [12] (incorrectly cited as “Adams et al.”), ChatGPT-4o, when asked to identify bones in unlabeled foot images, achieved correct labeling rates of 27%, 57%, and 0% for three different image prompts. Sallam et al. [20] (misattributed to “Alimova and Barch”) showed that, in clinical chemistry questions, ChatGPT-3.5's performance was notably lower than that of newer models (like ChatGPT-4) and often below 70% for complex medical questions. In the study by Arun et al. [13], the customized model Anatbuddy could "potentially outperform" ChatGPT on "factual accuracy and relevance" for anatomical queries, thus implying a ChatGPT-3.5 performance of less than 70% (Table 7).

**Table 7:** Summary of Gemini’s responses to the third query

Study	Key points
Bolgova et al. [7]	ChatGPT-3.5 provided accurate answers to 44.1% of anatomy questions. The performance varied by topic.
Mantzou et al. [9]	Only 50% of the answers were rated as "good quality". There was significant variability and inconsistency in the quality of answers over time.
Shamith et al. [12]	When asked to identify bones in unlabeled foot images, ChatGPT (version 4o, untrained) showed low labeling rates for three different image prompts.
Sallam et al. [20]	ChatGPT-3.5's performance was notably lower than that of newer models (like GPT-4) and often below 70% for complex medical questions.
Arun et al. [13]	Anatbuddy could "potentially outperform" ChatGPT on "factual accuracy and relevance for specific anatomical queries".

## Discussion

Regarding the first query, ChatGPT successfully identified and accurately summarized all five [1, 7-10] (100%) required studies. Among the retrieved studies, in only one case [9] ChatGPT’s performance (especially ChatGPT-4’s) was 75.6%, while in all other cases this performance was considerably lower. The platform also mentioned the review by Chytas et al. [1], which demonstrated that, in general, ChatGPT’s performance in anatomy questions was weak, while, in comparison with other AI platforms, this performance was equal or worse. Thus, ChatGPT’s self-assessment could be considered accurate and strict, given that it did not list papers that highlighted its superiority to other large language models.

Gemini successfully identified all five [7,9,11-13] (100%) required studies, but accurately summarized only two [7,9] (40%) of them. The studies by Bolgova et al. [7] and Mantzou et al. [9] were accurately summarized, with correct numerical data and valid conclusions. However, Gemini presented false outcomes of the study by Mavrych et al. [11], stating that no single model is superior to others. However, the study included specific numerical results and demonstrated that ChatGPT-4 was superior to all comparators (ChatGPT-3.5, ChatGPT-3.5 Turbo, Copilot, PaLM, Bard, and Gemini) in terms of answering anatomy questions. It can be noted that, in this study, ChatGPT-4 outperformed, among others, Gemini, but this fact was not pointed out. Also, although Gemini captured the conclusions of the paper by Shamith et al. [12], specific numerical data were not mentioned. These data showed that ChatGPT-4o’s performance was poor, given that the correct labeling rates for three prompts were 27%, 57%, and 0%, respectively. In the study by Arun et al. [13], Anatbuddy was significantly superior to ChatGPT-3.5 in terms of factual accuracy alone. However, Gemini stated that Anatbuddy was better than ChatGPT-3.5 in terms of both factual accuracy and relevance, while percentages of performance were not mentioned.

Regarding the second query, ChatGPT correctly identified and accurately summarized only one [11] (20%) of the five required studies. Four [14-17] retrieved studies were irrelevant to anatomy. The paper by Mavrych et al. [17] concerned neuroscience questions but lacked data distinguishing anatomy from physiology or neurological disorders. Also, the study by Al-Sharif et al. [14] contained only patients' oculoplastics-related clinical questions mentioning anatomical structures (such as eyelids) without testing anatomical knowledge. Regarding the study by Bolgova et al. [16] about biochemistry questions, ChatGPT falsely stated that these questions, being preclinical, had anatomy-related content. On the other hand, Gemini successfully identified three [5,18,19] (60%) of the five required studies. Of note, the platform hallucinated a publication entitled “Exploring the Performance of ChatGPT and Other AI Tools in Medical Education: A Systematic Review,” authored by Wang et al. in 2024. Moreover, the paper by Sallam et al. [20] was incorrectly mentioned, because it concerned clinical chemistry. Gemini unsuccessfully tried to “justify” its choice, stating that the comparative performance across various AI tools in clinical chemistry is highly relevant to their potential in anatomy. From the three [5, 18, 19] (60%) correctly identified studies, two [5, 19] (40%) were accurately summarized. Gemini captured the outline of the study by Al-Khater [18], but without giving percentages. Although it was stated that ChatGPT provided more accurate answers than Gemini, their quantitative difference (100% versus 60%) was not mentioned.

Concerning the third query, ChatGPT correctly detected three [21,22,24] (60%) of the five required studies and accurately summarized two [21,22] (40%) of them. More specifically, the platform included the research by Suárez et al. [23], which was about clinical endodontics, as well as the paper by Siebielec et al. [25], which involved medical examinations concerning several disciplines, apart from anatomy (although ChatGPT stated that anatomy was also examined). The study by Pavone et al. [24], although correctly identified, was inadequately summarized. Although ChatGPT mentioned that the performance in anatomical questions was below expectations, the percentage (50%) was not specified. Interestingly, the three [21, 22, 24] correctly detected studies showed performance much lower than the required 70% (and were found as low as 20%). This consistent reporting of performance lower than 70% suggests strict self-assessment. On the other hand, although Gemini correctly detected four [7, 9, 12, 13] (80%) of the five required studies, all five studies were previously mentioned, despite the relevant instruction. This fact indicates Gemini’s limited flexibility in finding literature about this topic. The platform accurately summarized three [7, 9, 12] (60%) of the five required studies. The study by Arun et al. [13] was again incorrectly summarized (with the aforementioned mistakes), while the irrelevant study by Sallam et al. [20] was again listed.

Overall, both platforms performed excellently only when they were simply asked to detect studies about ChatGPT’s ability to answer anatomy questions. ChatGPT conducted a strict self-evaluation and particularly struggled to locate and summarize comparative studies, while it performed better with studies containing numerical data about anatomy scores. Its performance was poor in the second query and moderate in the third one. Gemini showed difficulty in accurately summarizing findings across all three queries and was inflexible in detecting a variety of studies. Also, it falsely stated the authors in six cases [5, 9, 12, 18-20], while in one case, it hallucinated a publication. Of note, across all three queries, in total, four cases of inaccurate information provided by Gemini were against ChatGPT, indicating a bias. Similar to ChatGPT, Gemini particularly struggled to locate and outline comparative studies but performed generally better (Table 1).

Our study has limitations. The assessment of ChatGPT and Gemini was not based on a scoring grid and took place on a specific day and at a specific time. The creation of a quantitative tool for the evaluation of the performance of AI platforms in finding and summarizing literature could provide more robust data in the future. Furthermore, if the two AI tools are tested in other scientific domains or at different timepoints, their performance may vary. It is expected that the continuous AI development may enhance ChatGPT and Gemini’s potential to play a more significant role in facilitating anatomy researchers.

## Conclusions

ChatGPT and Gemini provided reliable responses only when they were simply asked to detect studies about ChatGPT’s performance in anatomy questions. Both platforms particularly struggled to locate and outline comparative studies, while Gemini performed generally better, yet its performance cannot be considered satisfactory. ChatGPT conducted a strict self-evaluation and did not hallucinate. Gemini was inflexible in finding a variety of publications, while it often hallucinated and exhibited bias against ChatGPT. Ongoing evolution may enhance ChatGPT and Gemini’s potential to essentially contribute to anatomical research.
